# Selection of Suitable Reference Genes for Quantitative Real-time PCR in *Sapium sebiferum*

**DOI:** 10.3389/fpls.2017.00637

**Published:** 2017-05-04

**Authors:** Xue Chen, Yingji Mao, Shengwei Huang, Jun Ni, Weili Lu, Jinyan Hou, Yuting Wang, Weiwei Zhao, Minghao Li, Qiaojian Wang, Lifang Wu

**Affiliations:** ^1^Key Laboratory of Ion Beam Bioengineering and Bioenergy Forest Research Center of State Forestry Administration, Hefei Institutes of Physical Science, Chinese Academy of SciencesHefei, China; ^2^Institute of Technical Biology & Agriculture Engineering, Science Island Branch of Graduate School, University of Science and Technology of ChinaHefei, China; ^3^School of Pharmacy, Anhui Medical UniversityHefei, China; ^4^The Sericultural Research Institute, Anhui Academy of Agricultural ScienceHefei, China; ^5^School of Forestry and Landscape Architecture, Anhui Agricultural UniversityHefei, China

**Keywords:** Chinese tallow (*Sapium sebiferum*), qRT-PCR, reference genes, gene expression, sucrose and cold stress

## Abstract

Chinese tallow (*Sapium sebiferum* L.) is a promising landscape and bioenergy plant. Measuring gene expression by quantitative real-time polymerase chain reaction (qRT-PCR) can provide valuable information on gene function. Stably expressed reference genes for normalization are a prerequisite for ensuring the accuracy of the target gene expression level among different samples. However, the reference genes in Chinese tallow have not been systematically validated. In this study, 12 candidate reference genes (*18S, GAPDH, UBQ, RPS15, SAND, TIP41, 60S, ACT7, PDF2, APT, TBP*, and *TUB*) were investigated with qRT-PCR in 18 samples, including those from different tissues, from plants treated with sucrose and cold stresses. The data were calculated with four common algorithms, geNorm, BestKeeper, NormFinder, and the delta cycle threshold (ΔCt). *TIP41* and *GAPDH* were the most stable for the tissue-specific experiment, *GAPDH* and *60S* for cold treatment, and *GAPDH* and *UBQ* for sucrose stresses, while the least stable genes were *60S, TIP41*, and *18S* respectively. The comprehensive results showed *APT, GAPDH*, and *UBQ* to be the top-ranked stable genes across all the samples. The stability of *60S* was the lowest during all experiments. These selected reference genes were further validated by comparing the expression profiles of the chalcone synthase gene in Chinese tallow in different samples. The results will help to improve the accuracy of gene expression studies in Chinese tallow.

## Introduction

Chinese tallow (*Sapium sebiferum* L.), which belongs to the *Euphorbiaceae* family is native to eastern Asia (Esser, 2002). It has been cultured for biofuel production and as an ornamental plant (Jeffrey and Padley, 1991). The fruits produce a highly saturated fatty acid in the tallow layer and highly unsaturated oil in the seed (Bolley and McCormack, 1950; Boldor et al., 2010). Tallow is applied for manufacturing candles, soap, cloth, and fuel, while the seed's oil can be used for making varnishes and native paints (Brooks et al., 1987; Jeffrey and Padley, 1991). It is estimated that the tree produces 4,700 l of oil ha^−1^ year^−1^. The average commercial yields far exceed those of traditional oil seed crops (Boldor et al., 2010). Nowadays, the tree is extensively propagated for ornamental purposes. With a moderate autumn chill, it can turn flaming red, plum purple, yellow orange, or mixed colors (Gao et al., 2016). Color is one of the most important characters of ornamental plants and directly affects their ornamental and economic value; creating novelty colors has been the pursuit of plant breeders (Zhao and Tao, 2015). Anthocyanins are water-soluble flavonoids that accumulate in vacuoles, which are responsible for plant pigments. Increasing and reducing anthocyanin content are likely to make the color change (Chalker-Scott, 1999; Winkel-Shirley, 2001). Moreover, anthocyanin biosynthesis is a response to biotic and abiotic stressors, including drought, temperature extremes, nitrogen and phosphorus deficiencies, wounding, bacterial, and fungal infections (Chalker-Scott, 1999). Therefore, anthocyanins are usually considered to be a stress symptom and enhance plant tolerance to the stress factors.

It has long been suggested that sugars are involved in anthocyanin synthesis in the reproductive organ or vegetative tissues (Mita et al., 1997; Baier et al., 2004). In *Vitis vinifera* cells, the production of anthocyanin was increased in the sucrose-containing medium, which is quite similar to *Arabidopsis thaliana* (Gollop et al., 2001; Lloyd and Zakhleniuk, 2004; Solfanelli et al., 2006). The sugar signal associated with anthocyanin accumulation is intimately linked to several signaling factors such as light and hormones, but the mechanisms are not well-understood (Das et al., 2012). Moderate low-temperature stress has been demonstrated as an important factor in increasing anthocyanin in *Arabidopsis*, maize (*Zea mays*), sorghum (*Sorghum bicolor*), and apple (*Malus domestica*) (Shichijo et al., 1993; Graham, 1998; Chalker-Scott, 1999; Rodriguez et al., 2014; Wang N. et al., 2016). In *Arabidopsis*, cold stress (4°C) induced the accumulation of anthocyanin in the leaves and stems (Leyva et al., 1995). Low temperature also induced anthocyanin accumulation in petunia (*Petunia hybrida*) flowers (Shvarts et al., 1997). In Chinese tallow, we also found that cold stress can induce anthocyanin biosynthesis in the leaves (Data not shown). However, the mechanism by which low-temperature stress induces anthocyanin accumulation in Chinese tallow remains unclear.

To promote gene function studies in Chinese tallow, accurately quantifying key genes expression will facilitate a better understanding of gene function in anthocyanin biosynthesis. Techniques such as Northern blot (Kevil et al., 1997), *in situ* hybridization (Ogilvie et al., 1990), RNase protection assay (Carey et al., 2013), serial analysis of gene expression (SAGE) (Velculescu et al., 1997), or quantitative real-time PCR (qRT-PCR) are often used to quantify gene expression (Kevil et al., 1997). Nowadays, qRT-PCR has become the preferred method due to its high sensitivity, specificity, and accuracy in detecting the target gene expression (Bustin et al., 2005). Moreover, qRT-PCR is fast, easy to use, and highly reproducible. It requires only a minimal amount of RNA and prevents the use of radioactivity (Radonic et al., 2004). The prerequisite for reliable gene expression analysis by qRT-PCR is the selection of appropriate reference genes, which can minimize the variations caused by it (Huggett et al., 2005). The reference genes are normally housekeeping genes, which are stably expressed in various samples across different experimental conditions or treatments (Thellin et al., 1999, 2009). In past decades, a series of reference genes, such as β-actin *(ACT)*, ubiquitin (*UBQ*), 18S ribosomal RNA (*18S rRNA*), and glyceraldehyde-3-phoshate dehydrogenase (*GAPDH*), have been widely used for gene expression analysis in plants (Bustin, 2002; Kim et al., 2003; Brunner et al., 2004; Czechowski et al., 2005). However, no reference genes are universal across different species and experimental conditions. For example, *GAPDH* has been regarded as a stable reference gene in coffee (*Coffea*) (Goulao et al., 2012), bermudagrass (*Cynodon dactylon*) (Chen et al., 2015), and physic nut (*Jatropha curcas*) (Zhang et al., 2013) under a series of experimental conditions. Nevertheless, *GAPDH* was the least stable reference gene in petunia (Mallona et al., 2010), tea plant (*Camellia sinensis*) (Wu et al., 2016), peach (*Prunus persica*) (Tong et al., 2009), and papaya (*Carica papaya*) (Zhu et al., 2012). Therefore, the selection of appropriate reference genes under different experimental conditions is essential for improving the accuracy and reliability of qRT-PCR. Typically, a set of candidate reference genes is evaluated in a pilot experiment with several statistical algorithms, such as geNorm (Vandesompele et al., 2002), BestKeeper (Pfaffl et al., 2004), NormFinder (Andersen et al., 2004), and the delta cycle threshold (ΔCt) (Silver et al., 2006) to identify the most suitable reference genes for qRT-PCR analysis.

Until now, no systematic validation of reference genes has been performed in Chinese tallow (Wang Y. et al., 2016). Therefore, we evaluated the expression stability of 12 genes (*18S, GAPDH, UBQ, RPS15, SAND, TIP41, 60S, ACT7, PDF2, APT, TBP*, and *TUB*) among the tissues of the seedlings and under sucrose and low- temperature stress treatments by qRT-PCR. To validate the reliability of the reference genes, the expression levels of the chalcone synthase gene in Chinese tallow (*SsCHS*) were analyzed. Chalcone synthase (*CHS*, EC 2.3.1.74) is a key enzyme in the first step for the biosynthesis of flavonoid and anthocyanin pigments in plants. It yields naringenin chalcone by condensing one p-coumaroyl-COA and three malonyl-CoA. In plants, *CHS* is activated by a wide range of environmental and developmental stimuli. Many studies have shown that the *CHS* gene's expression can be induced by light/UV light and in response to circadian clock, phytopathogens, elicitors or wounding, resulting in the enhanced production of flavonoids. In *Arabidopsis*, cold stress and sucrose can both directly induce *AtCHS* expression in leaves after treatment (Leyva et al., 1995). *SsCHS* is a homolog of the *CHS* gene from *Arabidopsis*. Thus, it would be expected to have similar expression patterns to *AtCHS* under abiotic and biotic stress conditions. Our results give an accurate and suitable reference gene selection for gene expression normalization, providing basic results for future gene expression studies on Chinese tallow tree.

## Materials and methods

### Plant materials and stress treatments

The seeds of Chinese tallow were collected from the experimental field at the Hefei Institutes of Physical Science (31°54′N, 117°10′E) of the Chinese Academy of Sciences (CASHIPS) located in Hefei, Anhui Province. Seeds were pre-treated with NaOH for 2 h to scrub the outer cellular tallow layer surrounding the seed coat, then stored in sand at room temperature for 3 months for germination (Li et al., 2011). Germinated seeds were transferred into the growth chamber at 25 ± 2°C under a 14/10 h (light/dark cycle) photoperiod with an irradiance of 40 μmol m^−2^ s^−1^ provided by cool white fluorescent lamps (Philips, India). Each group of three pots of 3-week-old seedlings with consensus growth status was selected and treated with sucrose and low temperature, respectively. For the low temperature treatment, seedlings were transferred into 4°C with the rest of the growing conditions being the same. For the sucrose treatment, 200 mM of sucrose (Sangon, China) solution was directly applied to the leaves. The leaves were collected at 0, 3, 6, 12, 24, 48, and 72 h after treatment according to the experiments conducted in *Arabidopsis* (Leyva et al., 1995; Solfanelli et al., 2006). The tissue samples from the root, stem, cotyledon, young leaf, and mature leaf were collected from the 3-week-old Chinese tallow seedlings. All samples were immediately frozen in liquid nitrogen and stored at −80°C for RNA extraction. Three biological replicates were collected for each sample.

### Total RNA extraction and cDNA synthesis

Total RNA was extracted using the E.Z.N.A.® Plant RNA Kit (Omega Bio-tec, USA) according to the manufacturer's protocol. The quantity and quality of the RNA were analyzed by 1.2% (w/v) agarose gel electrophoresis and a ScanDrop® Spectrophotometer (Analytik Jena AG, Jena, Germany). cDNA was synthesized by the *TransScript*® RT Reagent Kit (TransGen Biotech, Peking, China) according to the manufacturer's protocol. The cDNA was diluted 10-fold with RNAase-free water for qRT-PCR.

### Primer design and qRT-PCR analysis

The qRT-PCR primers (Table 1) were designed using the software Primer Premier 5. To verify the sequences of the reference genes, all the amplicons of the 12 candidate genes were analyzed by electrophoresis and sequencing. Each PCR reaction contained 10 μL of TransStart Top Green qPCR SuperMix (TransGen Biotech, Peking, China), 200 nM of each primer, and 2 μL of diluted cDNA in a total volume of 20 μL. No template controls (NTC) had water instead of cDNA as a template. The qRT-PCR reactions were performed with the qTOWER 2.0 Real-time PCR Detection System (Analytik Jena AG, Jena, Germany). The reactions were conducted under the following conditions: 30 s at 94°C for the initial denaturation, followed by 45 cycles of 5 s at 94°C, 15 s at the optimal temperature for each primer pair (Table 1), and 10 s at 72°C for PCR amplification. After 45 cycles, the dissociation curve was determined to confirm the specificity of each primer again by heating the product from 60 to 95°C. The normalized reporter (Rn) threshold was automatically selected to obtain the cycle threshold (Ct) values. The amplification efficiency of each set of primers was tested prior to the expression studies and calculated as E = −1 + 10 (−1/slope) (Pfaffl, 2001), where the slope was derived from a standard curve generated by five-fold serial dilutions of cDNA obtained from young leaves.

**Table 1:** **Genes description, amplification length, and PCR efficiency for *Sapium sebiferum* RGs selection**.

**Genes abbreviation**	**Description**	**Genbank number accession**	***Arabidopsis*** **ortholog locus**	**Primer sequences(5′–3′) Forward/Reverse**	**Amplification length (bp)**	**Efficiency (%)**	***R*****^2^**
*APT*	Adenine Phosphoribosyl transferase	KY656692	AT1G27450	CTGAGCCTGGAATGCTTTAT	159	1.89	0.992
				AATCTGATGGGAGTGACTTG			
*RPS15*	40s Ribosomal protein S	KY656693	AT1G04270	GAACCAGTCCGAACTCATCTT	184	1.88	1.000
				CACCAATACCAGGTCTACCAT			
*GAPDH*	Glyceraldehyde 3-phosphate dehydrogenase	KY656694	AT1G13440	AAGGGTGGTGCCAAGAAAGTC	149	1.88	0.997
				TTCGCAAGAGGAGCAAGACAG			
*TUB*	Tubulin beta chain	KY656695	AT1G20010	ATCTTGAACCTGGCACTAT	82	2.03	0.994
				TGCCCAAAGACGAAGTTAT			
*TBP*	TATA-box binding protein	KY656696	AT1G55520	ATTGGCAGCAAGGAAGTAT	154	1.93	0.997
				ACTTGAGAAAGCACCATGA			
*SAND*	SAND family protein	KY656697	AT2G28390	AATTAACAGTCCGCAACAGC	136	1.93	0.996
				GACCCAACAGAGTAGAACAT			
*60S*	60S ribosomal protein	KY656698	AT5G65220	CACTTTCGTCGGTAGTAATG	150	2.04	0.996
				TGAACTCGTTGCGTGCTGAT			
*UBQ*	Ubiquitin family	KY656699	AT4G05320	AAGGAACGGGTTGAGGAGAAA	131	1.81	0.975
				ACAAGATGAAGCACAGAGCCA			
*18S*	18S ribosomal RNA	KY656703	AT3G41768	TCTGCCCGTTGCTCTGATGAT	193	1.91	1.000
				CCTTGGATGTGGTAGCCGTTT			
*ACT7*	Actin 7	KY656700	AT5G09810	CAGTGTTTCCCAGTATTGTTG	165	1.87	1.000
				TTTCCATATCATCCCAGTTGC			
*TIP41*	tonoplast intrinsic protein 41	KY656701	AT4G34270	CTTCTCACAAGGGCTTCATC	98	1.93	0.991
				ACTGTCCCCAAACACCATCT			
*PDF2*	Protein phosphatase 2A	KY656702	AT1G13320	CGCCTGAACATAATAAGCAA	177	1.89	0.9945
				GAAGAACCCAACACCCAACT			
*SsCHS1*	Chalcone synthase	KY700827	AT5G13930	ATCTACGGACACCATCTCTTCT	116	1.87	0.996
				TTATCATCACCGATTTCCACCC			

### Data analysis of gene expression stability

Four algorithms, geNorm v. 3.5, BestKeeper, NormFinder, and ΔCt were used to rank the stability of the candidate reference genes. The pairwise variation (Vn/Vn+1) between two sequential normalization factors was obtained by geNorm software to determine the optimal number of reference genes needed for normalization. The recommended cut-o? threshold was 0.15, below which an additional control gene was not required for normalization. RefFinder (http://150.216.56.64/referencegene.php) was used to rank the stability of these candidates by comparing and integrating all four algorithms.

### Validation of reference genes

To validate the reliability of the reference genes, the expression levels of the *CHS* gene from Chinese tallow were analyzed via the top-ranking reference genes, as recommended by RefFinder, alone or with a combination for data normalization. In comparison, the least stable reference genes were also used. The statistical tests of gene expression data were calculated by one-way ANOVA. Statistical significance was considered at ^*^P < 0.05, ^**^P < 0.01, and ^***^P < 0.001.

## Results

### RNA quality, PCR specificity, and amplification efficiency of the candidate reference genes

To detect the RNA's quality and quantity, OD260, OD280, and OD230 were measured by the ScanDrop® Spectrophotometer. The OD260/OD280 ratios were between 1.8 and 2.0, and the OD260/OD230 ratios were higher than 2.0 (Table S1). This suggests high purity and freedom from organic contamination of the RNA sample. The purity and integrity of the RNA were also checked by agarose gel electrophoresis, and three RNA bands and no genomic DNA was observed (Figure S1). In this study, we selected 12 candidate reference genes through the homology search from the Chinese tallow transcriptome according to the stable reference genes reported in *Arabidopsis*. The primers for qRT-PCR were designed according to unigene sequences obtained from Chinese tallow tree transcriptome (Table 1). The sizes of all PCR products were from 82 to 193 bp (Table 1). All primer pairs showed a single major peak in the melting curve analysis (Figure S2) and a single band at the expected size by 2% agarose gel electrophoresis (Figure S3). No signal or band was detected in the negative control. The amplification efficiency of the primers varied from 0.81 to 1.07, and the correlation coefficients (*R*^2^) of the standard curve ranged from 0.9753 to 0.9999 (Table 1).

### Ct value distribution and expression profile of the 12 reference genes

**Figure 1:**
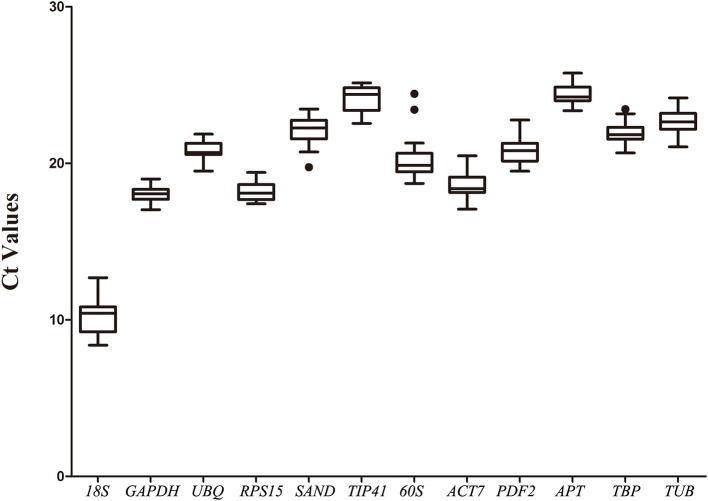
**Expression levels of 12 candidate reference genes across all experimental samples**. The box graph indicates the interquartile range, the median, and maximum/minimum values. Dots indicate outliers.

The expression levels of the 12 selected reference genes were confirmed with the threshold cycle values. All qRT-PCR assays were performed on three biological replicates. Most of the genes' Ct values varied from 17 to 25 (Figure 1). The *18S* gene showed the highest expression level in different samples, with the lowest Ct values being from 8.39 ± 0.46 to 12.7 ± 0.43. The *APT* gene showed the lowest expression level in the root, with the highest Ct values of 25.76 ± 0.2 (Table S2).

### GeNorm analysis

**Figure 2:**
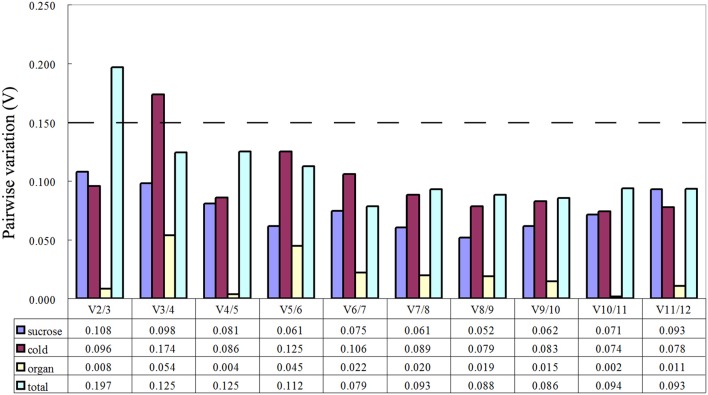
**Determination of the optimal number of reference genes for normalization by pairwise variation (V) using geNorm**. The pairwise variation (Vn/Vn+1) was calculated between normalization factors NFn and NFn+1, by geNorm to determine the optimal number of reference genes for qRT-PCR data normalization.

The geNorm software used in this study was used to evaluate the stability of the 12 candidate reference genes by calculating the gene expression stability (M). Genes with the lowest M value were considered to be most stable. As shown in Table 2, the M values of almost all genes were no more than the recommended threshold of 1.5. Cold stress treatment revealed that *GAPDH* and *60S* were the most stable genes. Sucrose stress results and tissue-specific experiments analysis showed that *GAPDH* and *ACT7* were the most stable genes. When the total samples were taken into account, the most stable genes were *RPS15* and *UBQ*, while *60S* was the least stable. The optimal number of reference gene analysis was based on average pairwise variation Vn/n+1 with a cut-off score of 0.15,below which the inclusion of an additional reference gene was not required. Of the three groups, V2/3 all had a score of < 0.15 (Figure 2). Notably, two reference genes were sufficient for accurate normalization. In consideration of the data from the whole groups, three genes were suitable for all samples in this study (Figure 2).

**Table 2:** **Stability of reference gene expression in each subset**.

**Experimental conditions**	**Reference gene**	**geNorm**		**Normfinder**		**Bestkeeper**			**Delta Ct**		**RefFinder**	
		**Stability**	**Rank**	**Stability**	**Rank**	**SD**	**CV**	**Rank**	**Stability**	**Rank**	**Stability**	**Rank**
Cold	*18S*	0.661	6	0.655	6	0.567	6.031	7	0.903	6	6.236	6
	*GAPDH*	0.268	1	0.308	1	0.430	2.348	2	0.705	1	1.189	1
	*UBQ*	0.625	5	0.779	9	0.613	2.919	8	0.966	10	7.746	8
	*RPS15*	0.740	9	0.443	4	0.464	2.485	4	0.752	4	4.899	5
	*SAND*	0.584	4	0.723	8	0.924	4.313	12	0.920	8	7.445	7
	*TIP41*	0.854	12	0.917	12	0.674	2.828	10	1.049	12	11.465	12
	*60S*	0.268	1	0.382	2	0.493	2.508	6	0.716	2	2.213	2
	*ACT7*	0.519	3	0.409	3	0.437	2.389	3	0.735	3	3.000	3
	*PDF2*	0.804	11	0.671	7	0.643	3.071	9	0.908	7	8.346	9
	*APT*	0.697	7	0.444	5	0.354	1.463	1	0.763	5	3.637	4
	*TBP*	0.771	10	0.802	10	0.486	2.229	5	0.995	11	8.612	10
	*TUB*	0.712	8	0.806	11	0.846	3.718	11	0.957	9	9.661	11
Sucrose	*18S*	0.699	12	1.110	12	0.572	5.440	10	1.178	12	11.465	12
	*GAPDH*	0.232	1	0.135	1	0.203	1.154	3	0.538	3	1.732	1
	*UBQ*	0.313	3	0.280	3	0.181	0.873	1	0.572	1	1.732	2
	*RPS15*	0.365	4	0.398	6	0.200	1.125	2	0.626	6	4.120	4
	*SAND*	0.489	8	0.517	9	0.495	2.210	9	0.687	9	8.739	9
	*TIP41*	0.603	11	0.749	11	0.670	2.743	12	0.888	11	11.242	11
	*60S*	0.547	10	0.659	10	0.663	3.309	11	0.794	10	10.241	10
	*ACT7*	0.232	1	0.223	2	0.314	1.726	4	0.573	2	2.000	3
	*PDF2*	0.459	7	0.501	8	0.475	2.338	8	0.680	8	7.737	8
	*APT*	0.398	5	0.341	5	0.360	1.489	6	0.608	5	5.233	6
	*TBP*	0.419	6	0.307	4	0.356	1.658	5	0.599	4	4.681	5
	*TUB*	0.508	9	0.413	7	0.442	1.959	7	0.647	7	7.454	7
Tissues	*18S*	0.606	11	0.480	7	0.620	5.520	11	0.760	10	9.593	10
	*GAPDH*	0.259	1	0.571	10	0.199	1.087	1	0.723	8	2.991	2
	*UBQ*	0.301	3	0.222	5	0.341	1.637	4	0.543	4	3.936	5
	*RPS15*	0.322	4	0.558	9	0.461	2.524	5	0.729	9	6.344	9
	*SAND*	0.414	7	0.495	8	0.307	1.369	2	0.677	7	5.292	8
	*TIP41*	0.470	8	0.061	1	0.483	2.017	7	0.493	1	2.736	1
	*60S*	0.821	12	1.799	12	1.886	8.743	12	1.828	12	12.000	12
	*ACT7*	0.259	1	0.090	3	0.544	2.781	9	0.546	5	3.409	3
	*PDF2*	0.388	6	0.381	6	0.317	1.477	3	0.594	6	5.045	7
	*APT*	0.525	9	0.065	2	0.462	1.837	6	0.506	2	3.834	4
	*TBP*	0.366	5	0.103	4	0.492	2.164	8	0.535	3	4.681	6
	*TUB*	0.566	10	0.587	11	0.580	2.572	10	0.766	11	10.488	11
Total	*18S*	0.890	11	0.986	11	0.840	8.159	11	1.180	11	11.000	11
	*GAPDH*	0.588	3	0.471	3	0.412	2.280	1	0.816	3	2.280	2
	*UBQ*	0.528	1	0.595	6	0.414	1.988	2	0.891	6	2.913	3
	*RPS15*	0.528	1	0.662	7	0.517	2.832	3	0.912	7	3.482	4
	*SAND*	0.784	9	0.717	9	0.696	3.157	10	0.990	9	9.240	9
	*TIP41*	0.828	10	0.834	10	0.686	2.849	9	1.064	10	9.740	10
	*60S*	0.953	12	1.103	12	1.010	4.975	12	1.266	12	12.000	12
	*ACT7*	0.679	6	0.411	2	0.629	3.384	7	0.812	2	3.600	5
	*PDF2*	0.626	4	0.590	5	0.653	3.137	8	0.888	5	5.318	7
	*APT*	0.639	5	0.327	1	0.521	2.131	4	0.773	1	2.115	1
	*TBP*	0.698	7	0.547	4	0.569	2.596	5	0.875	4	4.865	6
	*TUB*	0.741	8	0.698	8	0.619	2.734	6	0.967	8	7.445	8

### Normfinder analysis

Normfinder was employed to verify a more stable gene based on intra- group and inter- group variation. Similar to the GeNorm results, the M-value was also negatively correlated with its stability. In tissue-specific experiments, *TIP41* was the most stable gene. The cold-stress results and sucrose-stress treatment experiment both showed that *GAPDH* was the most stable gene. However, considering the M -value in all the above mentioned samples, the most stable reference gene was *APT* (Table 2).

### Bestkeeper algorithm

Bestkeeper ranks the genes' stability by computing the standard deviation (*SD*) value and coefficient of variance (CV). The lowest CV and SD (CV ± *SD*) means the most stable reference gene. SD values higher than 1 can be excluded (Table 2). *GAPDH* was the most stable gene for the tissue-specific experiment, while *UBQ* showed remarkably stable expression in the sucrose-stress treatment experiments. In the cold-stress treatment experiments, *APT* was identified as the most stable gene. *GAPDH* was the most stable gene for all the experiments.

### ΔCt method

The ΔCt method identifies the most stable genes by relative pair-wise comparisons. *UBQ* was the most stable gene in the sucrose treatment while *GAPDH* was the most stable in the cold treatment. *APT* was the most stable gene for the tissue-specific experiment and entire dataset (Table 2).

### Comprehensive stability analysis of reference ggenes by reffinder

Data generated by the four algorithms were further compared with the web-based comprehensive tool RefFinder. The results of the merged data revealed that the two best RGs for normalization were *GAPDH* and *60S* for the cold treatment, *GAPDH* and *UBQ* for the sucrose treatment, and *GAPDH* and *TIP41* for the tissue-specific experiments. Considering all the experiments, the combination of *UBQ*, G*APDH*, and *APT* as reference genes was the best choice. Based on the number of RGs suggested by geNorm and the ranking list suggested by RefFinder, the best combination of RGs for each treatment is shown in Table 3.

**Table 3:** **Best combination of RGs based on the geNorm and RefFinder**.

**Cold**		**Sucrose**		**Tissues**		**Total**	
**Most**	**Least**	**Most**	**Least**	**Most**	**Least**	**Most**	**Least**
*GAPDH*	*TIP41*	*GAPDH*	*18S*	*TIP41*	*60S*	*APT*	*60S*
*60S*		*UBQ*		*GAPDH*		*GAPDH*	
						*UBQ*	

### Expression analysis of *SsCHS* genes for reference gene validation

**Figure 3:**
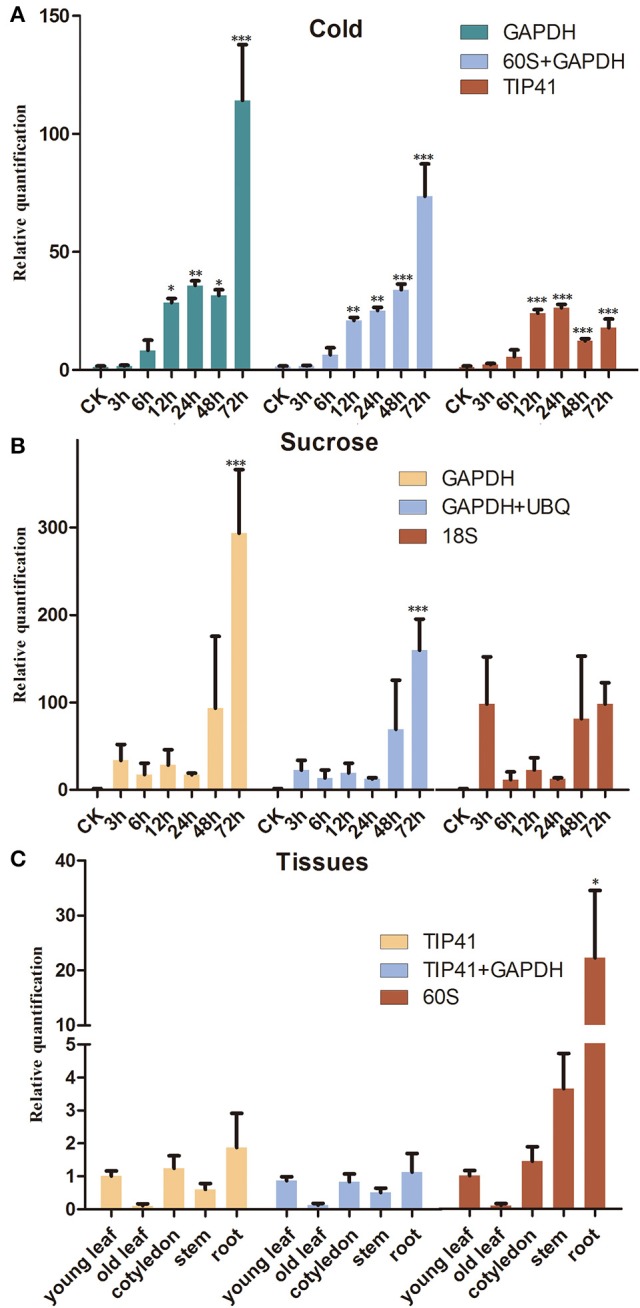
**Relative quantification of *SsCHS* expression using the validated reference gene(s). (A)** Leaves were collected from 3-week-old seedlings subjected to cold-stress after 0, 3, 6, 12, 24, 48, and 72 h of treatment. **(B)** Leaves were collected from 3-week-old seedlings subjected to sucrose-stress after 0, 3, 6, 12, 24, 48, and 72 h of treatment. **(C)** Different tissues were collected from 3-week-old seedlings. ^*^ indicates statistically significant (*p* < 0.05); ^**^Indicates greatly statistically significant (*p* < 0.01); ^***^Indicates greatly statistically significant (*p* < 0.001). The results are depicted as the mean ± *SD* (*n* = 3).

To confirm the validation of the reference genes, the *SsCHS* gene was examined under different experimental conditions with qRT-PCR. The results showed that similar to the expression pattern of *AtCHS* studied in *Arabidopsis*, the gene expression level of *SsCHS* was significantly induced by cold- and sucrose-stress treatments when normalized to the most stable reference genes. However, the gene expression values were quite different when different reference genes (most stable, a combination of most stable, least stable) were employed for normalization. Under the cold treatment, when the least stable gene, *TIP41*, was used for normalization, the maximum relative expression level of *SsCHS* was up-regulated 26.33 fold at 24 h, but when the most stable gene, *GAPDH*, was applied, the maximum relative expression level was up-regulated 114.22 at 72 h, which was more consistent with the maximum value of 91.02 at 72 h calculated with the combination of *60S* and *GAPDH* (Figure 3A). Under the sucrose treatment, when normalized with the most stable (*GAPDH*) and combination of reference genes (*GAPDH* and *UBQ*), the *SsCHS* expression patterns were basically the same as the level of the product continuous induced. However, the result was different without upregulation when normalized with the least stable gene *18S* (Figure 3B). For tissue-type analysis, when stable reference genes were used for normalization, *SsCHS* was expressed highly in the root, followed by cotyledon, young leaf, stem, and old leaf. However, the expression level of *SsCHS* in the root was overestimated when using the least stable reference gene (*60S*) for normalization (Figure 3C). These findings suggest that the choice of reliable reference genes is essential for the accurate normalization of target gene expression levels.

## Discussion

qRT-PCR is broadly used for the measurement of target gene expression. According to the “Minimum Information for publication of Quantitative real-time PCR Experiments” (MIQE) guidelines, using reference genes as the internal control is the most suitable normalization method for qRT-PCR analysis (Bustin et al., 2009). However, no single reference gene can be taken for quantifying the target genes expression levels under all conditions (Pfaffl et al., 2004; Huis et al., 2010). To obtain accurate results, it is essential to select suitable reference genes which were stably expressed in different tissues or under specific experiment conditions.

In this study, we report the systematic analysis of 12 reference genes, commonly used in other plant gene expression studies, in Chinese tallow under cold stress, sucrose stress, and in different tissues of the seedlings. The 12 genes were chosen in this paper based on previous studies, including both commonly used reference genes as well as new reference genes. In past studies, *18S* was taken as a reference gene for testing the expression of Diacylglycerol acyltransferases (*DGAT*s) in Chinese tallow (Wang Y. et al., 2016). *18S* is generated from a large, common precursor RNA (pre-rRNA). It has been exploited as a reference gene for a long time. However, in many species, such as *Oxytropis ochrocephala* Bunge and rubber tree, it was the least stable reference gene (Long et al., 2015; Zhuang et al., 2015). In addition, candidate genes showing high-level variation should be avoided as internal controls (Wang et al., 2015). Our results showed that the expression level of *18S* was too high and unstable compared with other reference genes, which is consistent with the results from papaya under many experimental conditions (Zhu et al., 2012).

In addition, four available methods (ΔCt, BestKeeper, NormFinder, and geNorm) were utilized for evaluating the stability of the expression levels of 12 candidate reference genes in five tissues and under two experimental treatments. The results showed that the least stable genes were nearly the same, while the most stable genes varied. For example, under the cold treatment, ΔCt, NormFinder, and geNorm all ranked *GAPDH* as the most stable gene, while BestKeeper ranked *APT* as the most stable. Then we used the RefFinder tool to compare and rank the 12 candidate reference genes based on the integrated results from each above mentioned program.

Many studies have proved that the accuracy of qRT-PCR can be improved by using more than one reference gene. In this study, two reference genes are needed for more accurate normalization under two stress treatment and in different plant tissues evaluated by geNorm software. The final ranking showed that *GAPDH* and *60S* were the most stable genes through the cold treatment. *GAPDH* and *UBQ* performed best as reference genes under the sucrose treatment. *GAPDH* has been demonstrated as a stable RG in physic nut, coffee and carrot (*Daucus carota*) under a series of experimental condition (Goulao et al., 2012; Zhang et al., 2013; Tian et al., 2015). However, it was the least stable reference gene in petunia, tea plant, peach, and papaya (Tong et al., 2009; Mallona et al., 2010; Zhu et al., 2012; Wu et al., 2016). *60S* was suggested as the most stable internal control in safflower (*Carthamus tinctorius*) and populus (*Populus euphratica*) under cold stress (Wang et al., 2014). *UBQ* was a stable reference gene proposed in different vegetative and reproductive tissues of sexual and apomictic *Boechera* (Pellino et al., 2011). It was also employed as internal controls for gene expression analysis in a wide variety of stages of longan somatic embryogenesis cultured under different temperatures (Lin and Lai, 2010). *TIP41* and *GAPDH* were selected as the stable reference genes in the tissues (young leaf, old leaf, stem, cotyledon, and root) of 3-week-old seedlings of Chinese tallow. *TIP41* was used as a reference gene for tissues (leaves, stems, cotyledons, hypocotyls, and roots) in peanut (*Arachis hypogaea*) (Chi et al., 2012) and bermudagrass leaves under heat stress (Chen et al., 2015).

To evaluate the suitability of the selected reference genes in this study, the expression levels of the *CHS* gene were examined in Chinese tallow. *CHS* encodes the first dedicated enzyme-*CHS* in the anthocyanin biosynthesis pathway, which catalyzes the stepwise condensation of three molecules of malonyl-CoA to one molecule of 4-coumaroyl-CoA to yield naringenin chalcone (Dooner et al., 1991). Anthocyanin is widely synthesized in seed plants to provide coloration, protection against various stresses, and components for cellular activities (Mol et al., 1998). In Chinese tallow, one of the most important colored tree species in China, anthocyanin is the primary substance that contributes to the leaf color (Feild et al., 2001). In addition, anthocyanin promotes the tree's ability to adapt to the environment. The study revealed that low-temperature-induced anthocyanin accumulation in red orange vesicles reached values eight times higher than those kept at 25°C after treatment for 75 days. Furthermore, qRT-PCR confirmed that the expression of *PAL, CHS, DFR*, and *UFGT* was strongly induced by low temperature, since the expression levels of all transcripts increased at least 40-fold compared with the control samples (Lo Piero et al., 2005). In *Arabidopsis*, sucrose treatment can strongly up-regulate the expression of key genes involved in anthocyanin biosynthetic pathways and anthocyanin content (Solfanelli et al., 2006).

Here, in Chinese tallow seedlings, *SsCHS* was remarkably continuously induced by the cold and sucrose treatments. For tissue-specific analysis, *SsCHS* was highly expressed in the root with a relatively lower expression level in old leaves when the most stable reference genes were used for normalization. Anthocyanins accumulated in non-photosynthetic tissues, were postulated to act as modulators of reactive oxygen signaling cascades involved in plant growth and development. These results conclusively provide the information required for selecting reliable reference genes in gene expression studies and ensure more accurate and reliable gene expression quantification in Chinese tallow.

## Author contributions

XC and YM conceived and designed the experiments. XC, YM, and JN performed the experiments. XC and YM analyzed the data. SH, WL, ML, YW, JH, and QW contributed reagents/materials. XC, JN, and WZ wrote the manuscript. LW edited the manuscript.

### Conflict of interest statement

The authors declare that the research was conducted in the absence of any commercial or financial relationships that could be construed as a potential conflict of interest.

## Supplementary material

Figure S1 **Agarose gel (1.2 %) electrophoresis of the RNA**.

Figure S2 **Melting curves of 12 reference genes showing single peaks**.

Figure S3 **Agarose gel (2%) electrophoresis showing amplification of a single PCR product of the expected size; M represents DL2000 DNA marker**.

Table S1 **RNA purity**.

Table S2 **Cycle threshold (Ct) values for twelve candidate reference genes**.
