# Supplementary figures and images for: Selection of Suitable Reference Genes for Quantitative Real-time PCR in Sapium sebiferum
Source: Front Plant Sci. 2017 May 4;8:637. doi: 10.3389/fpls.2017.00637 (PMC5415600; doi:10.3389/fpls.2017.00637)

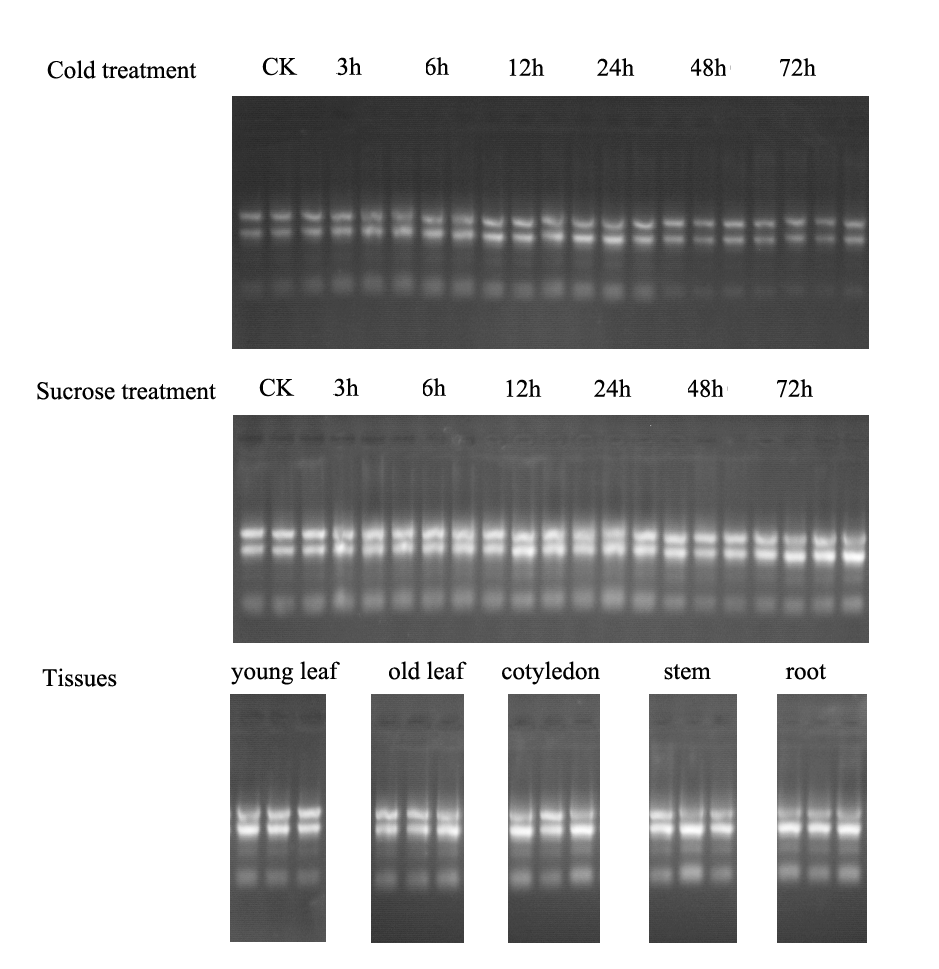

Supplement: Figure S1 — Agarose gel (1.2 %) electrophoresis of the RNA. [file Image1.TIF]

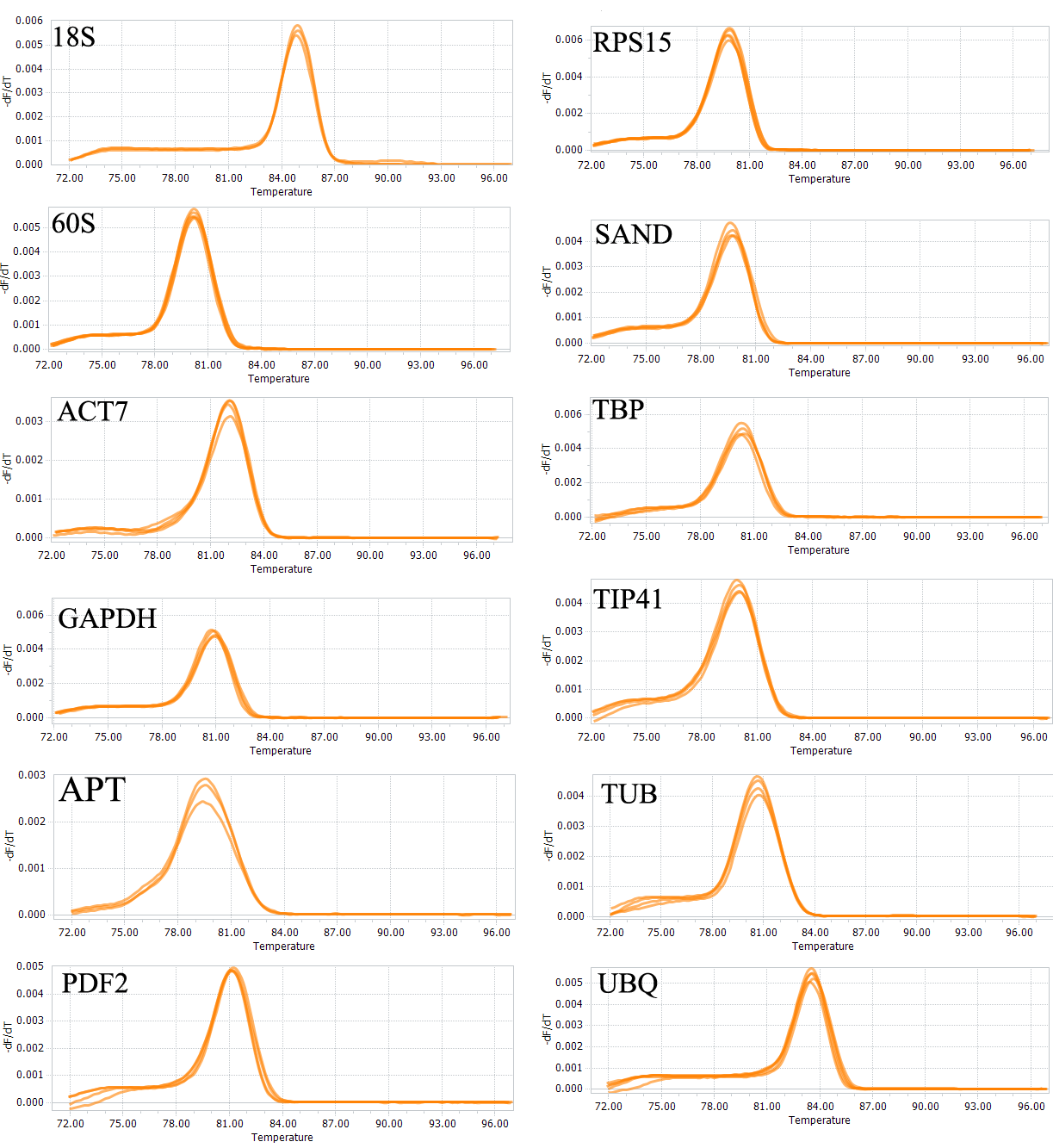

Supplement: Figure S2 — Melting curves of 12 reference genes showing single peaks. [file Image2.TIF]

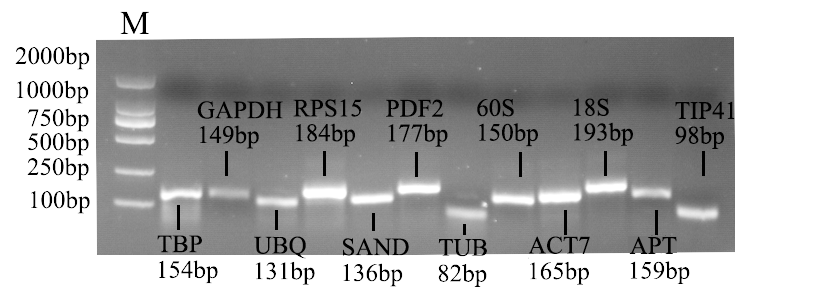

Supplement: Figure S3 — Agarose gel (2%) electrophoresis showing amplification of a single PCR product of the expected size; M represents DL2000 DNA marker. [file Image3.TIF]
